# Supplementary material for: PhyDOSE: Design of follow-up single-cell sequencing experiments of tumors
Source: PLoS Comput Biol. 2020 Oct 1;16(10):e1008240. doi: 10.1371/journal.pcbi.1008240 (PMC7553321; doi:10.1371/journal.pcbi.1008240)
Supplement: S1 Text — (PDF) [file pcbi.1008240.s001.pdf]

Supplementary Text — PhyDOSE: Design of Follow-up Single-cell Sequencing Experiments of Tumors

Leah Weber [1,*] Nuraini Aguse [1,*] Nicholas Chia [2,3] Mohammed El-Kebir [1,†]

[1]Dept. of Computer Science, University of Illinois at Urbana-Champaign, Urbana, IL 61801
[2]Microbiome Program, Center for Individualized Medicine, Mayo Clinic, Rochester, MN 55905
[3]Division of Surgical Research, Department of Surgery, Mayo Clinic, Rochester, MN 55905

[*]Shared first authorship; [†]Corresponding author: melkebir@illinois.edu

Contents

A Supplementary Text **2**
A.1 Complexity 2

B Supplementary Methods **4**
B.1 Finding the Minimal Distinguishing Feature Family Φ^* 4
B.2 Confidence Interval for k^* 4
B.3 Heuristic Power Calculation for Multiple Biospies 6
B.3.1 Integer Linear Program for Selecting Featurettes per Biopsy 7

C Supplementary Results **10**
C.1 Retrospective Analysis of an Acute Lymphoblastic Leukemia Patient 18

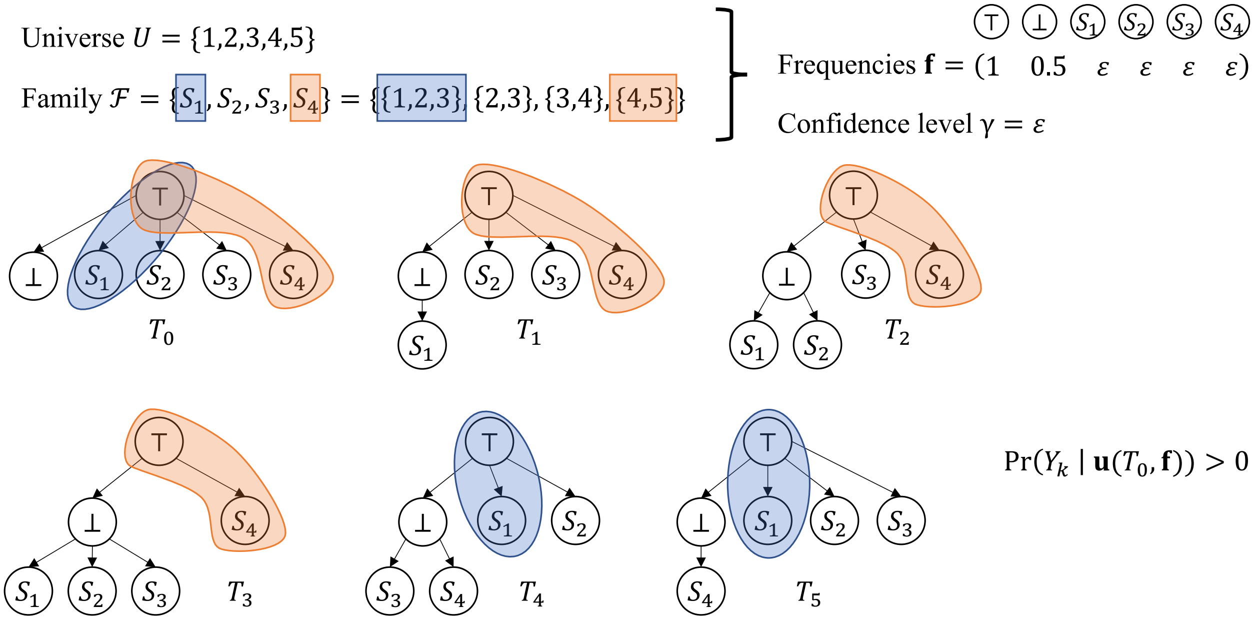

Fig A: **Reduction from Set Cover to T-SCS-PC**. Given a family $\mathcal{F}$ of subsets $\{S_1, \ldots, S_{|\mathcal{F}|}\}$ on a universe $U = \{1, \ldots, n\}$, we construct $n+1$ trees $\mathcal{T} = \{T_0, \ldots, T_n\}$ with mutations $\{\top, \bot, S_1, \ldots, S_{|\mathcal{F}|}\}$. We seek to distinguish T_0 from the remaining trees $\{T_1, \ldots, T_n\}$. The key concept captured by the reduction is that there is a cover of size k if and only if $\Pr(Y_k \mid \mathbf{u}(T_0, \mathbf{f}))$ is greater than 0. Here, S_1 and S_4 form a cover of size $k = 2$ of the universe U and the corresponding probability $\Pr(Y_k \mid \mathbf{u}(T_0, \mathbf{f}))$ is greater than 0.

A Supplementary Text

A.1 Complexity

Theorem 1. T-SCS-PC is NP-hard.

We prove the theorem using a polynomial-time reduction from the Set Cover problem, a known NP-hard problem [1].

Problem 1 (Set Cover)**.** Given a family $\mathcal{F}$ of subsets $\{S_1, \ldots, S_{|\mathcal{F}|}\}$ over a universe $U = \{1, \ldots, n\}$, find a cover $C \subseteq \mathcal{F}$ such that $\bigcup_{S \in C} S = U$ and C has minimum cardinality.

Specifically, we reduce a Set Cover instance $(\mathcal{F}, U)$ to an T-SCS-PC instance $(\mathcal{T}, T, \mathbf{f}, \gamma)$ as follows. The set $\mathcal{T} = \{T_0, \ldots, T_n\}$ includes one tree T_i for each element i in the universe U and an additional tree T_0. All trees in $\mathcal{T}$ have $|\mathcal{F}| + 2$ vertices, corresponding to subsets $\{S_1, \ldots, S_{|\mathcal{F}|}\}$ and two additional mutations $\{\top, \bot\}$. Each tree in $\mathcal{T}$ includes the edge $(\top, \bot)$. Additionally, if element $i \in U$ is absent from subset S_j then there is an edge $(\top, S_j)$ in tree T_i, otherwise T_i includes an edge $(\bot, S_j)$. Tree T_0 includes edges $(\top, S_j)$ for all subsets S_j. As for the frequencies $\mathbf{f}$, we set $f_\top = 1$, $f_\bot = 0.5$ and the remaining frequencies $f_{S_j} = \varepsilon$ for all subsets $S_j \in \mathcal{F}$. Moreover, we set the confidence level γ to ε as well. In the corresponding T-SCS-PC instance $(\mathcal{T}, T_0, \mathbf{f}, \varepsilon)$, the tree of interest is T_0. Fig. A shows an example.

The key idea is that as $\gamma = \varepsilon > 0$ is a small positive infinitesimal constant, this T-SCS-PC instance seeks the smallest number k^* of cells such that $\Pr(Y_{k*} \mid \mathbf{u}(T_0, \mathbf{f}))$ is non-zero. In particular, this number k^* of cells will only be achieved if there is a distinguishing feature Π of the same size k^*. By our reduction, there is a 1-1 correspondence between set covers of U and distinguishing features Π of T_0 with respect to $\{T_1, \ldots, T_n\}$. Specifically, a set cover C of size k corresponds to a distinguishing feature $\Pi(C)$ of the same size k, and vice versa. As such, we have the following lemma whose proof is in the supplement.

Lemma 1. Let $(\mathcal{T}, T_0, \mathbf{f}, \gamma = \varepsilon)$ be the T-SCS-PC instance corresponding to SET COVER instance $(U, \mathcal{F})$. A minimum cover has size k^* if and only if k^* is the smallest integer such that $\Pr(Y_{k^*} \mid \mathbf{u}(T_0, \mathbf{f})) \geq \gamma$.

Proof. $(\Rightarrow)$ Let C be a minimum cover of the SET COVER instance $(U, \mathcal{F})$. By the premise, we have that $|C| = k^*$. We start by showing that $\Pr(Y_{k^*} \mid \mathbf{u}(T_0, \mathbf{f})) \geq \gamma$ by constructing a distinguishing feature $\Pi(C)$ of T_0 where $|\Pi| = k^*$. Observe that for each subset S_j in C we have that $\{\top, S_j\}$ is a featurette of T_0. We define $\Pi(C)$ to be composed of featurettes $\{\top, S_j\}$ for all subsets $S_j \in C$. Thus, $|\Pi(C)| = k^*$. To show that $\Pi(C)$ is a distinguishing feature of T_0, it remains to show that at least one featurette $\tau \in \Pi(C)$ is absent in each tree in $\mathcal{T} \setminus T_0 = \{T_1, \ldots, T_n\}$. Consider any tree $T_i \neq T_0$. Since C is a cover, the element i of the universe U corresponding to tree T_i must be covered by some subset $S_j \in C$. This means that tree T_i contains the edge $(\bot, S_j)$, which means that the featurette $\{\top, S_j\}$ in $\Pi(C)$ is absent from T_i. Hence, $\Pi(C)$ is a distinguishing feature of T_0.

We now must show that $\Pr(Y_{k^*} \mid \mathbf{u}(T_0, \mathbf{f})) \geq \gamma$. We do so by focusing on distinguishing feature $\Pi(C)$. By construction of T_0 and $\mathbf{f}$, it follows from the sum condition (see Main Text) that each featurette $\{\top, S_j\}$ in $\Pi(C)$ has a clonal prevalence $u_j = \varepsilon$. This means that a SCS experiment of k^* cells where we only observe the k^* featurettes/clones has a probability that is strictly greater than 0. Therefore, $\Pr(Y_{k^*} \mid \mathbf{u}(T_0, \mathbf{f})) > 0$. Since ε is a small positive infinitesimal constant, we have that $\Pr(Y_{k^*} \mid \mathbf{u}(T_0, \mathbf{f})) \geq \gamma = \varepsilon$.

It remains to show that k^* is the smallest integer where $\Pr(Y_{k^*} \mid \mathbf{u}(T_0, \mathbf{f})) \geq \varepsilon$. Assume for a contradiction that the smallest integer k' where $\Pr(Y_{k'} \mid \mathbf{u}(T_0, \mathbf{f})) \geq \varepsilon$ is strictly smaller than k^*. This means that there exists a minimal distinguishing feature Π' of size at most k'. By definition Π' is composed of featurettes corresponding to root-to-vertex paths in T_0. Since Π' is minimal, it will not contain the featurette $\{\top, \bot\}$ as this featurette is present in all remaining trees $\{T_1, \ldots, T_n\}$. Thus, Π' is composed of featurettes of the form $\{\top, S_j\}$ where $S_j \in \mathcal{F}$. Since Π' is a distinguishing feature, no tree $T_i \in \{T_1, \ldots, T_n\}$ contains all featurettes of Π'. By construction of $\{T_1, \ldots, T_n\}$, this means that the subsets encoded in Π' form a cover of the universe U. Thus, there exists a cover with size strictly smaller than k^*, contradicting the premise. Therefore, k^* is indeed the smallest integer where $\Pr(Y_{k^*} \mid \mathbf{u}(T_0, \mathbf{f})) \geq \gamma = \varepsilon$.

$(\Leftarrow)$ Let k^* be the smallest integer such that $\Pr(Y_{k^*} \mid \mathbf{u}(T_0, \mathbf{f})) \geq \gamma = \varepsilon$. We start by showing that the size of a minimum distinguishing feature Π of T_0 has to be exactly k^*. Clearly, if $|\Pi| > k^*$ then $\Pr(Y_{k^*} \mid \mathbf{u}(T_0, \mathbf{f})) = 0$ as there exists no successful SCS experiment with k^* cells. On the other hand, if $|\Pi| < k^*$ then there exists a successful SCS experiment with $|\Pi|$ cells. In other words, $\Pr(Y_{|\Pi|} \mid \mathbf{u}(T_0, \mathbf{f})) \geq \varepsilon$. This contradicts that k^* is the smallest integer where $\Pr(Y_{|\Pi|} \mid \mathbf{u}(T_0, \mathbf{f})) \geq \varepsilon$. Hence, $|\Pi| = k^*$.

Consider a minimum distinguishing feature Π of T_0. By the previous argument, we know that $|\Pi| = k^*$. We will show that Π encodes a cover $C(\Pi)$ of U of size k^*. Since Π is minimal, it will not contain the featurette $\{\top, \bot\}$ of T_0 as this featurette is present in all remaining trees $\{T_1, \ldots, T_n\}$. Thus, Π is composed of k featurettes of the form $\{\top, S_j\}$ where $S_j \in \mathcal{F}$. Let $C(\Pi)$ be defined as the collection of subsets $S_j \in \mathcal{F}$ where $\{\top, S_j\}$ in Π. Since Π is a distinguishing feature, no tree $T_i \in \{T_1, \ldots, T_n\}$ contains all featurettes of Π. By construction of $\{T_1, \ldots, T_n\}$, this means that $C(\Pi)$ is a cover of size k of the universe U.

Finally, we must show that there exists no cover C' of U with size $|C'|$ strictly smaller than k^*. Suppose for a contradiction that such a cover C' exists. By construction, C' encodes a distinguishing feature $\Pi(C')$ composed of featurettes $\{\top, S_j\}$ for all subsets $S_j \in C'$. Thus, $|\Pi(C')| = |C'|$. To show that $\Pi(C')$ is a distinguishing feature of T_0, we must show that (i) all features $\tau \in \Pi(C')$ are present in T_0, and (ii) at least one featurette $\tau \in \Pi(C)$ is absent in each tree in $\mathcal{T} \setminus T_0 = \{T_1, \ldots, T_n\}$. Condition (i) holds by construction of $\Pi(C')$ and T_0, i.e. for each subset S_j in C' we have that $\{\top, S_j\}$ is a featurette of T_0. As for condition (ii), consider any tree $T_i \neq T_0$. Since C' is a cover, the element i of the universe U corresponding to tree T_i must be covered by some subset $S_j \in C'$. This means that tree T_i contains the edge $(\bot, S_j)$, which means that the featurette $\{\top, S_j\}$ in $\Pi(C')$ is absent from T_i. Hence, $\Pi(C')$ is a distinguishing feature of T_0. This in turn means that $\Pr(Y_{|\Pi(C')|} \mid \mathbf{u}(T_0, \mathbf{f})) > 0$. In other words, $\Pr(Y_{|\Pi(C')|} \mid \mathbf{u}(T_0, \mathbf{f})) \geq \gamma = \varepsilon$, thus contradicting the premise. Hence, minimum set covers of $(U, \mathcal{F})$ have cardinality k^*. □

The theorem follows from the above lemma, as the reduction to obtain $(\mathcal{T}, T_0, \mathbf{f}, \gamma = \varepsilon)$ from $(U, \mathcal{F})$ takes only polynomial time.

B Supplementary Methods

B.1 Finding the Minimal Distinguishing Feature Family Φ^*

To perform the calculation, it is necessary to first find the minimal distinguishing feature family Φ^*. Using similar ideas as in our hardness proof (Section A.1), we consider the reverse reduction from the problem of finding a minimal distinguishing feature to that of finding a minimum size set cover (Problem 1).

We define the universe $U = \{1, \ldots, m\}$ to be the set $\mathcal{T} \setminus \{T\} = \{T_1, \ldots, T_m\}$ of trees excluding the tree T for which we want to solve the T-SCS-PC problem. We define the family $\mathcal{F} = \{S_1, \ldots, S_n\}$ of subsets to correspond to the n featurettes present in T. Specifically, the subset S_j corresponding to featurette τ_j that is present in T is composed of elements $i \in U$ corresponding to trees T_i where τ_j is absent. The key idea is that S_j is indicating in which input trees featurette τ_j of T is absent. We note that $\mathcal{F}$ is a multi-set as distinct featurettes τ_j and $\tau_{j'}$ may be absent in the same set of trees, thus leading to $S_j = S_{j'}$ ((Main Text) Fig. 3b). There is a bijection between set covers of $(U, \mathcal{F})$ and distinguishing features of T. That is, each distinguishing feature $\Pi = \{\tau_1, \ldots, \tau_{|\Pi|}\}$ corresponds to the same-sized cover $\Pi(C)$ composed of subsets $\{S_1, \ldots, S_{|\Pi(C)|}\}$, and vice versa. In particular, a minimal distinguishing feature corresponds to a minimal set cover.

We used the following combinatorial algorithm to exhaustively enumerate all minimal distinguishing features of a given tree T. The algorithm generates all possible features Π of T that are of sizes $i = 1, \ldots, m$.

Algorithm 1: MINIMALDFF(T)

Input: Tree T
Output: Minimal distinguishing feature family Φ^*

```
m ← number of featurettes in T
Φ* ← ∅
for i ← 1 to m do
    for each feature Π containing i featurettes do
        if Π(T) and Π minimal w.r.t Φ* then
            Φ* ← Φ* ∪ {Π}
return Φ*
```

Since we enumerate features Π from size smallest to largest, we ensure that every feature $\Pi' \subsetneq \Pi$ was visited before it. Thus, if Π is minimal w.r.t Φ^*, it means that all features $\Pi' \subsetneq \Pi$ that were visited before it were not distinguishing. If Π is also a distinguishing feature of T, we add it to Φ^*. Hence, Φ^* contains all minimal distinguishing features of T. (Main Text) Fig. 3b shows an example.

B.2 Confidence Interval for k^*

The cancer cell fractions (CCFs) $\mathbf{f}$ of mutations are a key input to PhyDOSE. Specifically, the CCF f_i of a mutation i is estimated from the numbers of variant and reference reads spanning the mutation locus and additional copy number information. Uncertainty in these frequency estimates will impact the design of the follow-up single-cell sequencing experiment. Following a standard approach [2, 3, 4], we construct a confidence interval $[f_i^-, f_i^+]$ of the CCF of mutation i by modeling the number of variants reads as a binomial or beta-binomial distribution using a specified level of confidence $\alpha \in [0, 1]$. Here α is the probability that

the true parameter estimate f_i falls outside the constructed confidence interval $[f_i^-, f_i^+]$. Thus, the input to PhyDOSE is composed of two frequency vectors $\mathbf{f}^-$ and $\mathbf{f}^+$.

Recall that PhyDOSE performs a power calculation on the number of cells to sequence such that the probability of successfully observing a distinguishing feature of a given tree $T \in \mathcal{T}$ is at least a given confidence level γ. This probability is modeled as the tail probability of the multinomial distribution where the probabilities are obtained from the clonal prevalence $\mathbf{u}(T, \mathbf{f}) = [u_i]$ of each featurette/clone i in the distinguishing features. These clonal prevalences are uniquely determined by the given tree T and frequencies f under the infinite sites assumption as

$$u_i = f_i - \sum_{j \in \delta_T(i)} f_j \qquad\qquad \forall i \in [n], \tag{1}$$

where $\delta_T(i)$ is the set of children of the node where mutation i was introduced [2]. In other words, to obtain non-negative clonal prevalences, it must hold that $f_i \geq \sum_{j \in \delta_T(i)} f_j$ for all mutations i. Thus, given a candidate set $\mathcal{T}$, a tree $T \in \mathcal{T}$ and a range $[\mathbf{f}^-, \mathbf{f}^+]$ for the cancer cell fractions, we reduce the problem of finding the k^* confidence interval to one of finding two assignments $[\hat{\mathbf{f}}^-, \hat{\mathbf{f}}^+]$ of frequencies within the given range $[\mathbf{f}^-, \mathbf{f}^+]$ of frequencies that can be utilized directly in the PhyDOSE power calculation. Specifically, $\hat{\mathbf{f}}^-$ corresponds to the theoretical minimum number $k^*_-(T)$ and $\hat{\mathbf{f}}^+$ corresponds to theoretical maximum number $k^*_+(T)$ of single cells for each tree T. Here we formally state two problems to be solved in order to obtain the $[k^*_-(T), k^*_+(T)]$ confidence interval.

Problem 2. For a given tree T in candidate set $\mathcal{T}$, frequencies $[\mathbf{f}^-, \mathbf{f}^+]$ and confidence level γ, find frequencies $\hat{\mathbf{f}}^-$ such that (i) $f_i^- \leq \hat{f}_i^- \leq f_i^+$ for each mutation i and (ii) $k_*^- = \mathrm{argmin}_k \Pr(Y_k \mid \mathbf{u}(T, \hat{\mathbf{f}}^-)) \geq \gamma$ is minimum.

Problem 3. For a given tree T in candidate set $\mathcal{T}$, frequencies $[\mathbf{f}^-, \mathbf{f}^+]$ and confidence level γ, find frequencies $\hat{\mathbf{f}}^+$ such that (i) $f_i^- \leq \hat{f}_i^+ \leq f_i^+$ for each mutation i and (ii) $k_*^+ = \mathrm{argmin}_k \Pr(Y_k \mid \mathbf{u}(T, \hat{\mathbf{f}}^+)) \geq \gamma$ is maximum.

We solve both problems heuristically using the following two simplifications. First, for the sake of a clear exposition, we assume that the distinguishing feature family $\Phi(T, \mathcal{T} \setminus \{T\})$ is composed of a single distinguishing feature Π — we will describe below how this assumption can be relaxed. Second, rather than jointly considering all featurettes, we optimize the featurette in the distinguishing feature that will yield the smallest clonal prevalence from the assigned frequency values $\hat{\mathbf{f}}^-$ and $\hat{\mathbf{f}}^+$. The rationale is that the multinomial tail probability is closely approximated by the binomial tail probability utilizing the minimum probability featurette in the distinguishing feature [5]. In the following, we develop two linear programs to heuristically find the frequencies $\hat{\mathbf{f}}^-$ and $\hat{\mathbf{f}}^+$.

Recall that trees under the infinite sites assumption exhibit a one-to-one correspondence between mutations and clones. In addition, recall that the distinguishing feature Π is composed of a set of distinguishing featurettes/clones. Let $X(\Pi)$ be the set of mutations that identify these clones. To find values $\hat{\mathbf{f}}^-$, we use a linear program with an objective to maximize the minimum clonal prevalence among all featurettes in the

distinguishing feature.

$$\max z \tag{2}$$

$$\text{s.t. } \hat{f_i^-} \geq \sum_{v_j \in \delta(v_i)} \hat{f_j^-}, \qquad \forall i \in [n], \tag{3}$$

$$z \leq \hat{f_i^-} - \sum_{j \in \delta_T(i)} \hat{f_j^-}, \qquad \forall i \in X(\Pi), \tag{4}$$

$$f_i^- \leq \hat{f_i^-} \leq f_i^+, \qquad \forall i \in [n], \tag{5}$$

$$z \geq 0, \tag{6}$$

where $\delta_T(i)$ is the set of children of the node where mutation i was introduced. The first constraint ensures the frequencies $\hat{\mathbf{f}}^-$ yields a non-negative clonal prevalence for all clones in tree T. The second constraint is to facilitate the objective to maximize the minimum clonal prevalence of the featurettes in the distinguishing feature. The last constraint ensures the assigned frequencies $\hat{\mathbf{f}}^-$ are within the specified frequency confidence interval.

In the second linear program, we find values $\hat{\mathbf{f}}^+$ to heuristically solve Problem 3 with an objective to maximize the minimum clone prevalence among all featurettes in the distinguishing feature.

$$\min z \tag{7}$$

$$\text{s.t. } \hat{f_i^+} \geq \sum_{v_j \in \delta(v_i)} \hat{f_j^-}, \qquad \forall i \in [n], \tag{8}$$

$$z \geq \sum_{j \in \delta_T(k)} \hat{f_j^+} - \hat{f_k^+}, \qquad \forall k \in \Pi_j, \tag{9}$$

$$f_i^- \leq \hat{f_i}^+ \leq f_i^+, \qquad \forall i \in [n], \tag{10}$$

$$z \geq 0. \tag{11}$$

The first and last constraints are the same as in the previous linear program. In the second constraint, we ensure the problem remains convex by switching the sign of the clonal prevalence values and setting z to be greater than the maximum negative clonal prevalence of all featurettes in the distinguishing feature. We use the `lpSolve`[1] R package solve these linear programs within the `phydoser` R package.

When a tree has multiple distinguishing features, we solve the two linear programs for each distinguishing feature $\Pi \in \Phi^*$ returning frequencies $\hat{\mathbf{f}}^-$ and $\hat{\mathbf{f}}^+$ that achieve the maximum objective value of the respective linear programs. This is because it is only necessary to observe a single distinguishing feature for an experiment to be successful. Thus, the probability calculation will be dominated by the distinguishing feature where the smallest featurette clonal prevalence is maximum over the smallest featurette clonal prevalence rates of all distinguishing features.

The goal of the confidence interval is to consider uncertainty in the frequency parameter estimates when determining k^*. Therefore, we opt to construct the k^* confidence interval conservatively. Specifically, we first construct a confidence interval for each tree $T \in \mathcal{T}$. Then, we take the confidence interval that corresponds to the tree with the maximum upper end of the confidence interval as the confidence interval for k^*.

B.3 Heuristic Power Calculation for Multiple Biospies

Given multiple biopsies from the same tumor, the T-MUL-SCS-PC seeks the numbers $\mathbf{k}^* = [k_1^*, \ldots, k_b^*]$ of cells to sequence from each biopsy such that $||\mathbf{k}^*||_1$ is minimum. Here, we develop a heuristic because

[1] `http://lpsolve.sourceforge.net/5.5/`

the exact approach presented in the main text does not scale. Given a frequency matrix $F \in [0,1]^{b \times n}$, confidence level γ and the minimal distinguishing feature family Φ^* of a tree T with respect to candidate trees $\mathcal{T}$, the heuristic proceeds in two steps in order to select a subset of clones to detect in each biopsy that together form a distinguishing feature in Φ^* and achieve the smallest total number of cells with confidence level γ.

First, we perform a power calculation at confidence level $\gamma^{1/b}$ for each biopsy and subset of featurettes present in Φ^*. Due to independence, a confidence of $\gamma^{1/b}$ in each biopsy will achieve the overall confidence of γ, i.e. $\prod_{p=1}^{b} \gamma^{1/b} = \gamma$. To define the desired family of subsets of featurettes, let $N = \bigcup_{\Pi \in \Phi^*} \Pi$ be the set of featurettes present in the distinguishing features of Φ^*, and let $\{\Psi(1), \ldots, \Psi(2^{|N|})\}$ be the powerset of featurettes N (Fig. B). We define $Z_{k,\Psi(i),p}$ as the event of observing the set $\Psi(i)$ of featurettes/clones in an SCS experiment with k cells from biopsy p. Using the multinomial power calculation outlined previously, we compute the minimum number k_{ip} of cells required to observe the set $\Psi(i)$ from biopsy p such that the probability of success is at least $\gamma^{1/|b|}$. That is,

$$k_{ip} = \operatorname*{argmin}_{k \in \mathbb{N}} \Pr(Z_{k,\Psi(i),p} \mid u(T, \mathbf{f}_p)) \geq \gamma^{1/|b|}. \tag{12}$$

Note that for the index i where the corresponding featurette subset $\Psi(i)$ equals the empty set $\emptyset$, we have that $k_{ip} = 0$ as $\Pr(Z_{0,\emptyset,p} \mid u(T, \mathbf{f}_p)) = 1$ for all biopsies p.

Second, given the number k_{ip} for each featurette subset $i \in [2^{|N|}]$ and biopsy p, we now want to pick a subset of featurettes $\Psi(\sigma(1)), \ldots, \Psi(\sigma(b))$ for each biopsy p that together cover at least one distinguishing feature in Φ^* and have the minimum total number $\sum_{p=1}^{b} k_{\sigma(p),p}$ of cells. In Section B.3.1, we provide a mixed integer linear program (MILP) to solve this problem. To solve the MUL-SCS-PC problem, we take the per biopsy maximum across all solutions to T-MUL-SCS-PC for each $T \in \mathcal{T}$.

This approach is a heuristic in two ways. First, the heuristic does not account for events where we observe a distinguishing feature in ways other than the selected featurettes per biopsy. As such, the heuristic might overestimate the total number of cells that are needed for the specified confidence γ, as we show in Fig. B. Second, when solving the MUL-SCS-PC, we only consider a single optimal solution for each $T \in \mathcal{T}$. Indeed, it may be possible to find a solution $\mathbf{k}^*$ with a smaller L_1 norm if we consider the entire set of feasible solutions for each tree.

B.3.1 Integer Linear Program for Selecting Featurettes per Biopsy

Let the minimal distinguishing feature family Φ^* be composed of distinguishing features $\{\Pi_1, \ldots, \Pi_{|\Phi^*|}\}$. The featurettes in Φ^* correspond to $N = \{\tau_1, \ldots, \tau_{|N|}\}$. The powerset of featurettes N equals $\{\Psi(1), \ldots, \Psi(2^{|N|})\}$. Recall that k_{ip} indicates the minimum number of cells required to observe the featurette set $\Psi(i)$ from biopsy p such that the probability of success is at least $\gamma^{1/|b|}$. Given these values, we develop an integer linear program (ILP) whose objective is to select a subset $\Psi(\sigma(p))$ of featurettes for each biopsy p such that the total number $\sum_{p=1}^{b} k_{\sigma(p),p}$ of cells is minimum and the selected featurettes cover at least one distinguishing feature in Φ^*. To accomplish this, we need variables and constraints to model the following.

- Assignment of a featurette subset to each biopsy.

  Binary variables $\mathbf{x} \in \{0,1\}^{2^{|N|} \times b}$ indicate the assignment of featurette subsets to biopsies, modeled by (14).

- Coverage of a distinguishing feature.

  The key constraint is that the selected featurette subsets cover at least one distinguishing feature in Φ^*. To model this constraint, we need several helper variables and constraints.

We begin with binary variables $\mathbf{r} \in \{0,1\}^{2^{|N|}}$, which indicate that feature subset $\Psi(i)$ is assigned to at least one biopsy. This is modeled by constraints (15) and (16).

We introduce binary variables $\mathbf{z} \in \{0,1\}^{|N|}$, which indicate the coverage of featurettes τ_ℓ present in Φ^*. To that end, for a featurette $\tau_\ell \in N$, let $\Psi^{-1}(\ell)$ be the set of indices i corresponding to featurette subsets $\Psi(i)$ that contain featurette τ_ℓ. Constraints (17) and (18) use variables $\mathbf{r}$ to model that $z_\ell = 1$ if and only if a featurette subset containing τ_ℓ has been selected.

Next, binary variables $\mathbf{y} \in \{0,1\}^{|\Phi^*|}$ indicate the coverage of distinguishing features by $\mathbf{x}$. To that end, we use variables $\mathbf{z}$ in constraints (19) and (20).

Finally, constraint (21) uses binary variables $\mathbf{y}$ to require that at least one distinguishing feature of Φ^* is covered.

$$
\begin{aligned}
\min\ & \sum_{i=1}^{2^{|N|}} \sum_{p=1}^{b} k_{ip} x_{ip} && && (13)\\
\text{s.t.}\ & \sum_{i=1}^{2^{|N|}} x_{ip} = 1\,, && \forall p \in [b], && (14)\\
& r_i \le \sum_{p=1}^{b} x_{ip}\,, && \forall i \in [2^{|N|}], && (15)\\
& r_i \ge x_{ip}\,, && \forall i \in [2^{|N|}], p \in [b], && (16)\\
& z_\ell \le \sum_{i \in \Psi^{-1}(\ell)} r_i\,, && \forall \ell \in [N], && (17)\\
& z_\ell \ge r_i\,, && \forall \ell \in [N], i \in \Psi^{-1}(\ell), && (18)\\
& y_j \ge \sum_{\tau_\ell \in \Pi_j} z_\ell - |\Pi_j| + 1\,, && \forall j \in [|\Phi^*|], && (19)\\
& y_j \le z_\ell\,, && \forall j \in [|\Phi^*|], \tau_\ell \in \Pi_j, && (20)\\
& \sum_{j=1}^{|\Phi^*|} y_j \ge 1\,, && && (21)\\
& x_{ip} \in \{0,1\}\,, && \forall i \in [2^N], p \in [b], && (22)\\
& r_i \in \{0,1\}, && \forall i \in [2^N], && (23)\\
& z_\ell \in \{0,1\}, && \forall \ell \in [N], && (24)\\
& y_j \in \{0,1\}, && \forall j \in [|\Phi^*|]. && (25)
\end{aligned}
$$

Note that we can relax the integrality constraints (23), (24) and (25) as this will be enforced by integrality of $\mathbf{x}$. Doing so will lead to a speed up. Using the `lpSolveAPI`[2] R package solve to this ILP for each tree T within the `phydoser` R package.

[2] `http://lpsolve.sourceforge.net/5.5/`

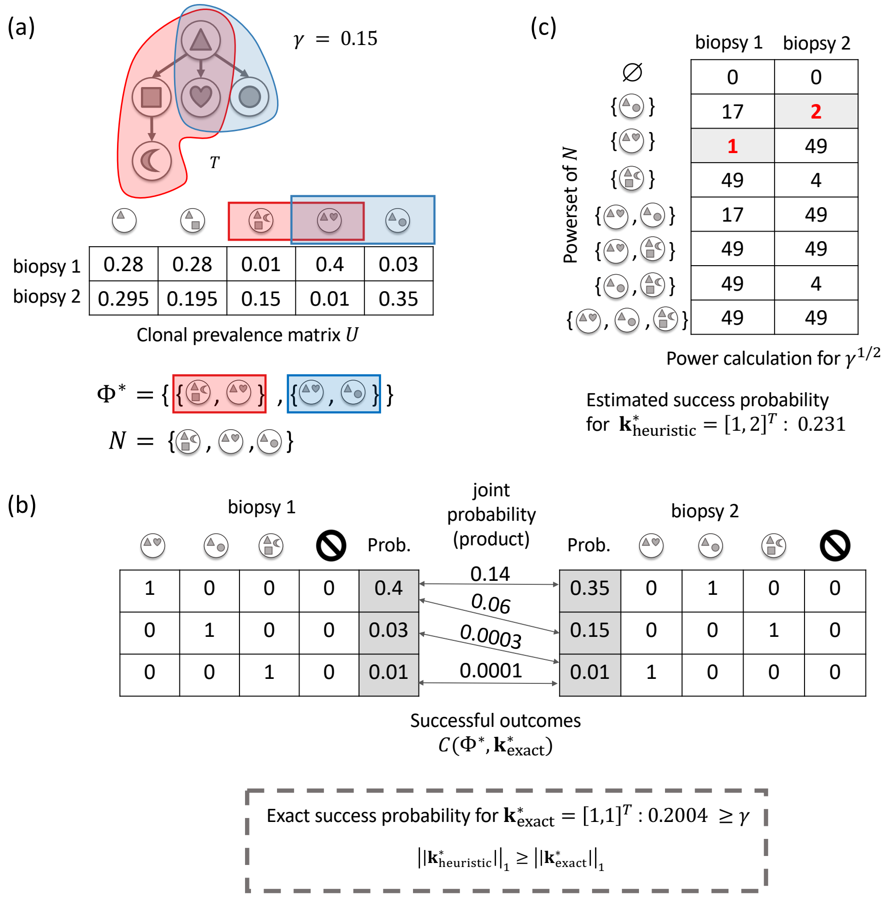

Fig B: **Solving the T-MUL-SCS-PC problem** (A) An example instance of the T-MUL-SCS-PC problem with $b = 2$ biopsies, where we are given a confidence level γ, a clonal prevalence matrix U and the minimal distinguishing feature family Φ^*. The set N of featurettes is the union of the distinguishing features in Φ^*. (B) For $k^*_{\text{exact}} = [1, 1]^T$ cells, there are four outcomes in the set $C(\Phi^*, k^*_{\text{exact}})$ of successful outcomes. The probability of each successful outcome is the joint probability of observing the required number of cells in each biopsy. Here, the probability $\Pr(Y_{k^*_{\text{exact}}} \mid U)$ of success equals 0.2004, which is greater than $\gamma = 0.15$. Moreover, $\Pr(Y_{[1,0]^T} \mid U) = \Pr(Y_{[0,1]^T} \mid U) = 0$ as each distinguishing feature has at least two featurettes. Hence, $k^*_{\text{exact}} = [1, 1]^T$ is the optimal solution to this T-MUL-SCS-PC problem instance. (C) The heuristic performs a power calculation at confidence level $\gamma^{1/b} = \gamma^{1/2}$ for each element of the powerset of N and each biopsy. Then, an ILP assigns a featurette set to each biopsy minimizing the total number of cells while covering at least one distinguishing feature. Here, the heuristic solution is $\mathbf{k}^*_{\text{heuristic}} = [1, 2]^T$, which is worse than the exact calculation.

C Supplementary Results

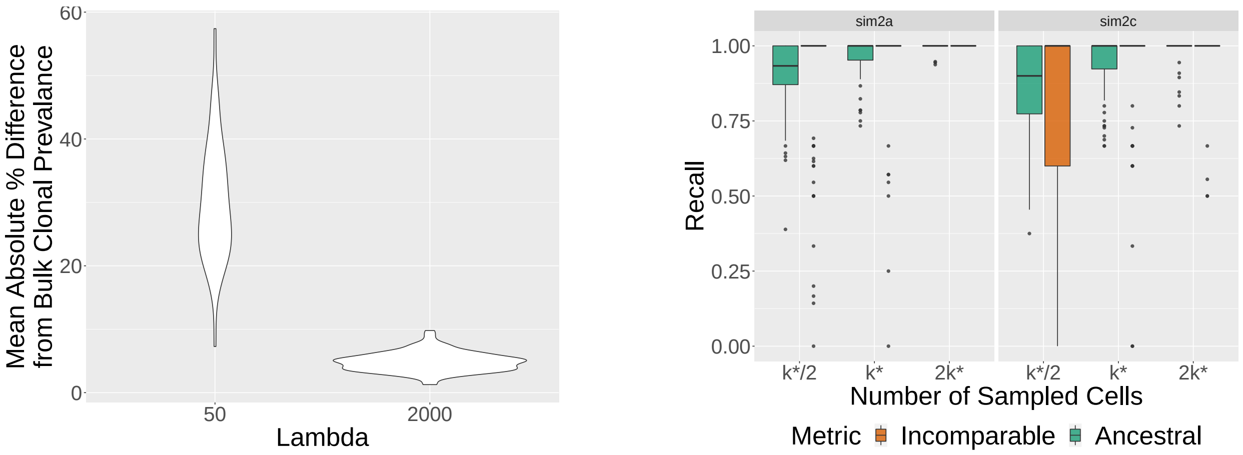

A B

Fig C: **PhyDOSE sensitivity analysis of clonal prevalence distortion** (A) The mean absolute percentage difference of the single cell clonal prevalence per replication from simulated bulk clonal prevalence at values of $\lambda \in \{50, 2000\}$. These values of λ resulted in mean absolute percentage difference of 5% and 20% respectively. (B) $\lambda = 2000$ was selected for sim2a/b but further sensitivity analysis was performed with $\lambda = 50$. The recall metrics are compared between sim2a at $\lambda = 2000$ and sim2c at $\lambda = 50$. (C) Recall metrics when inferring T^* with SPhyR [6] by randomly sampling $k^*/2, k^*, 2k^*$ simulated single cells.

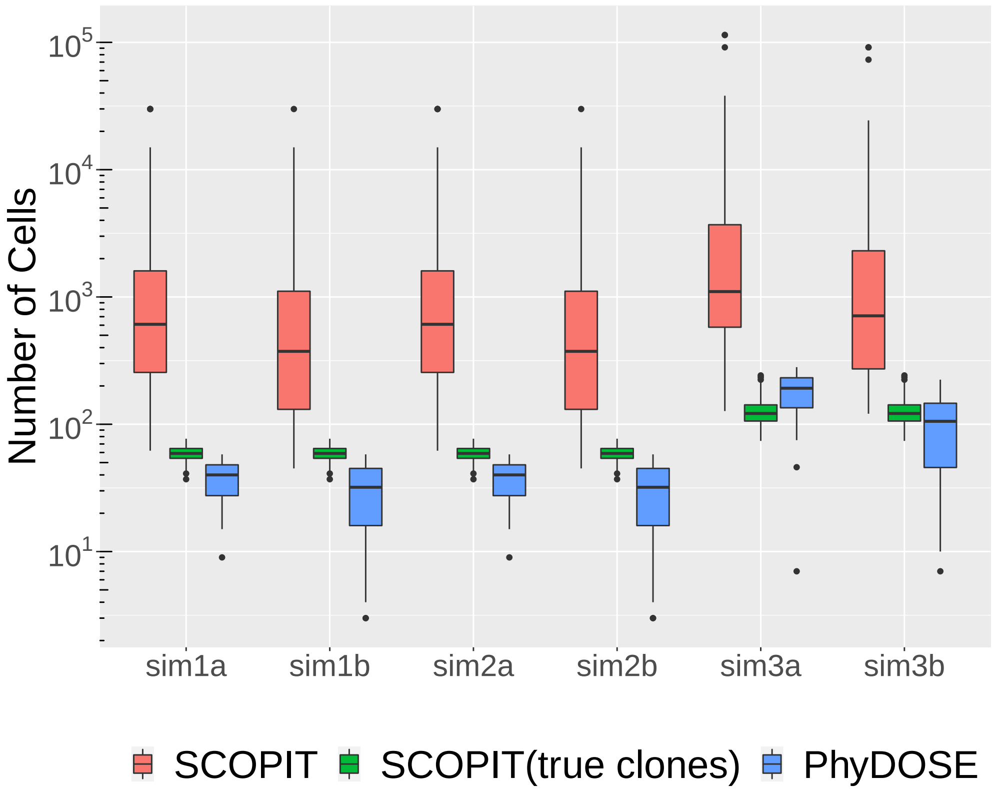

Fig D: **PhyDOSE comparison with SCOPIT** The distribution of the recommended number of cells to sequence per simulated replication determined by SCOPIT, SCOPIT using the true clones, and PhyDOSE.

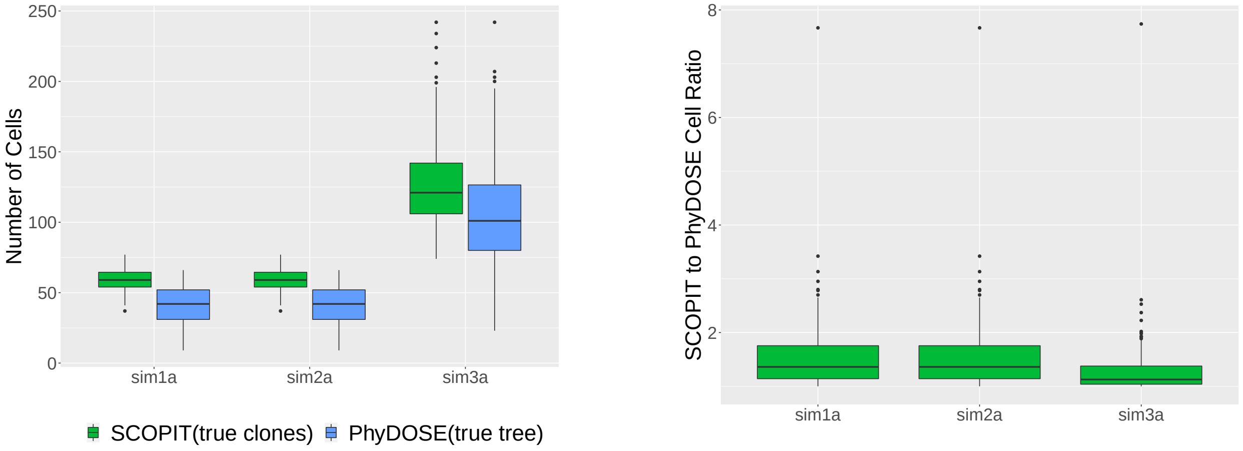

A B

Fig E: **PhyDOSE $k^*(T^*)$ comparison with SCOPIT (true clones)** (A) The distribution of the recommended number of cells to sequence per simulated replication determined by SCOPIT when given the ground truth clones and PhyDOSE $k^*(T^*)$. (B) The cell ratio per simulation between SCOPIT (true clones) and PhyDOSE $k^*(T^*)$

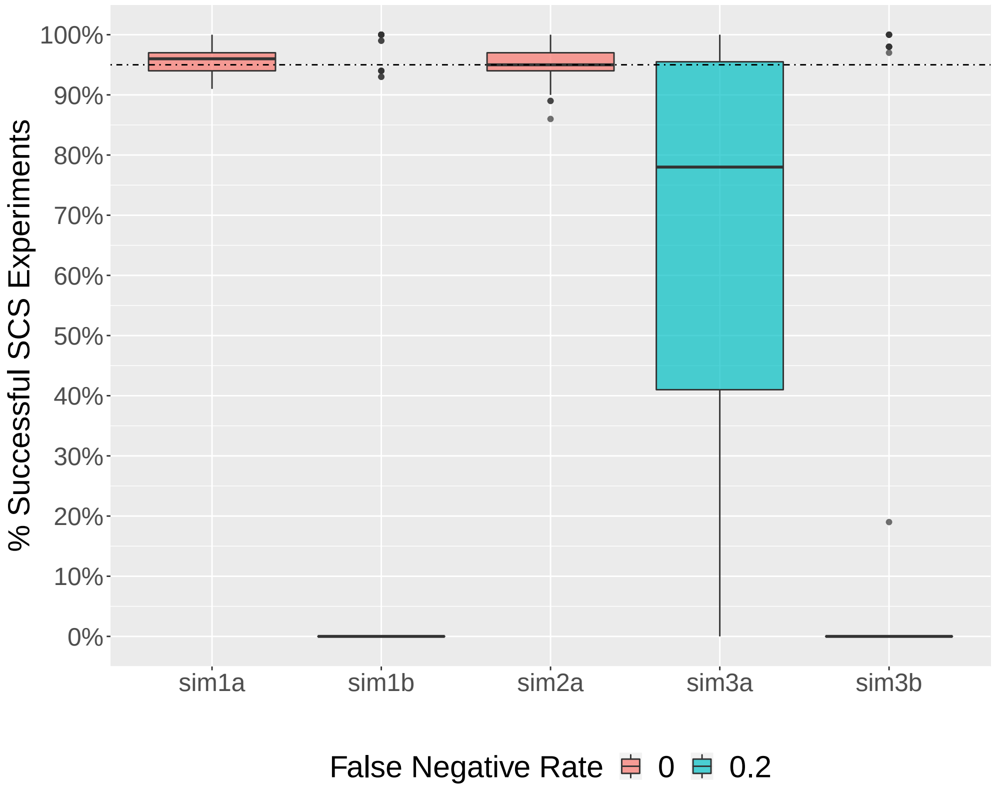

Fig F: **Percentage of *in silico* single-cell sequencing experiments correctly identifying T^* as the true tree utilizing PhyDOSE's k^* at γ =0.95 and $\beta \in \{0.0, 0.2\}$.** PhyDOSE has a high success rate in alignment with $\gamma = 0.95$ (dashed horizontal line) when utilizing the entire set $\mathcal{T}$ in condition a. However, the introduction of false negatives results in a reduction in success rate and greater variance. In the cases where $T^* \in \mathcal{T}$, the support metric was still able to successfully identify T^* as the true tree.

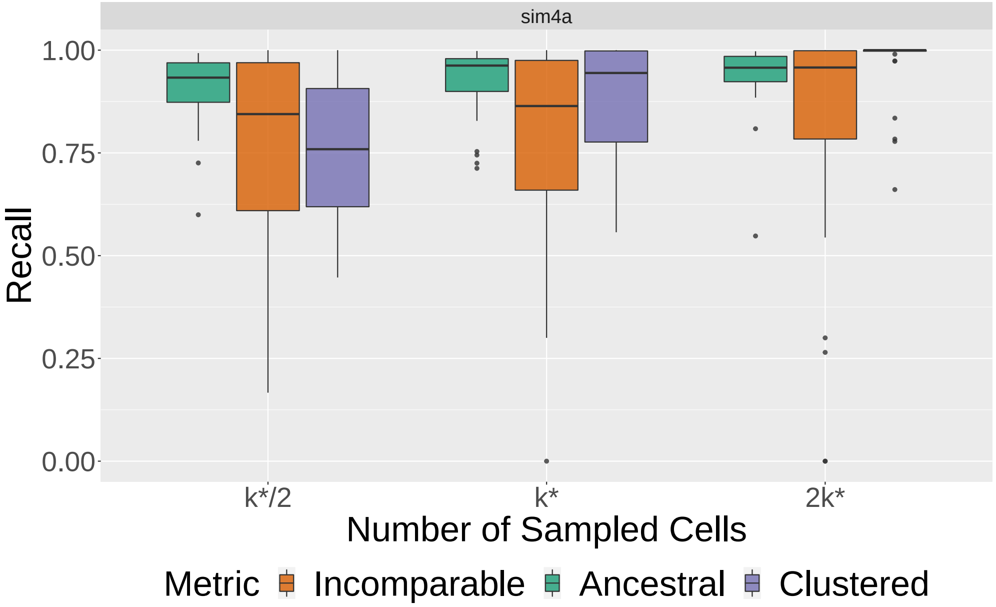

Fig G: **PhyDOSE performance with mutation clustering** Recall metrics when inferring T^* with SPhyR [6] by randomly sampling $k^*/2, k^*, 2k^*$ simulated single cells.

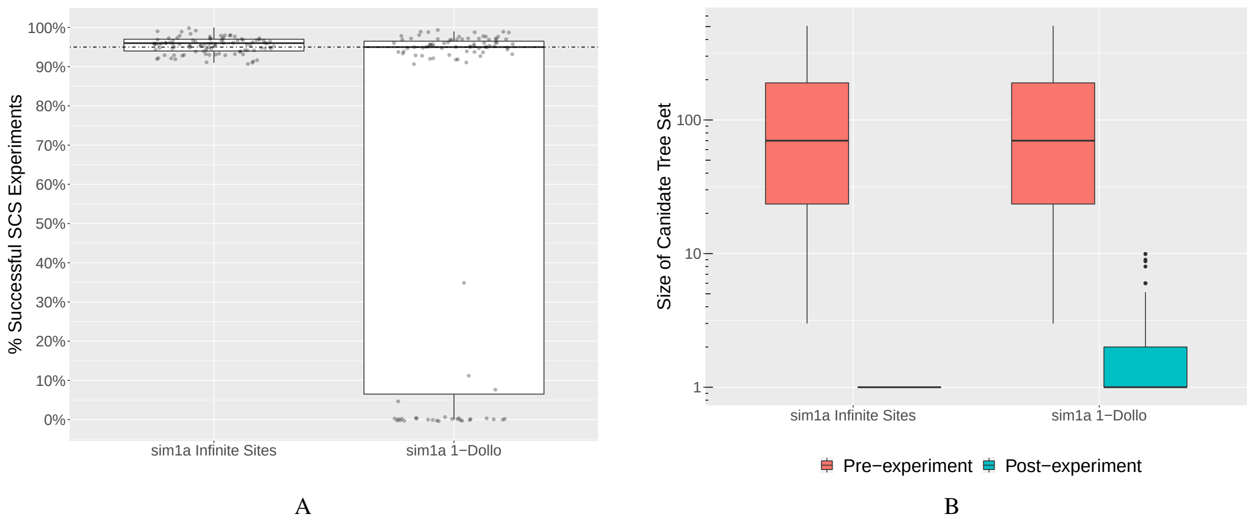

Fig H: **Assessment of PhyDOSE with respect to violations of the infinite sites assumption**. (A) Percentage of *in silico* single-cell sequencing experiments correctly identifying T^* as the true tree utilizing PhyDOSE's k^* at confidence level $\gamma = 0.95$ and false negative rate $\beta = 0$ under the infinite sites model and the 1-Dollo model. PhyDOSE has a high success rate in alignment with $\gamma = 0.95$ under both models of evolution. The variance in the success rate in the 1-Dollo model can be attributed to whether or not the distinguishing features are impacted by the mutation loss. When the distinguishing features are no longer distinguishing, then the experiment will be unsuccessful. (B) The average number of trees in the candidate set $\mathcal{T}$ pre and post execution of an experiment designed by PhyDOSE. The post-experiment candidate set $\mathcal{T}$ contains all trees T that yielded $\mathrm{support}(T) > 0$ during the experiment. Even in cases where the experiment does not uniquely identify T^* as the true tree, the size of the candidate set is still significantly reduced as compared to the input candidate set.

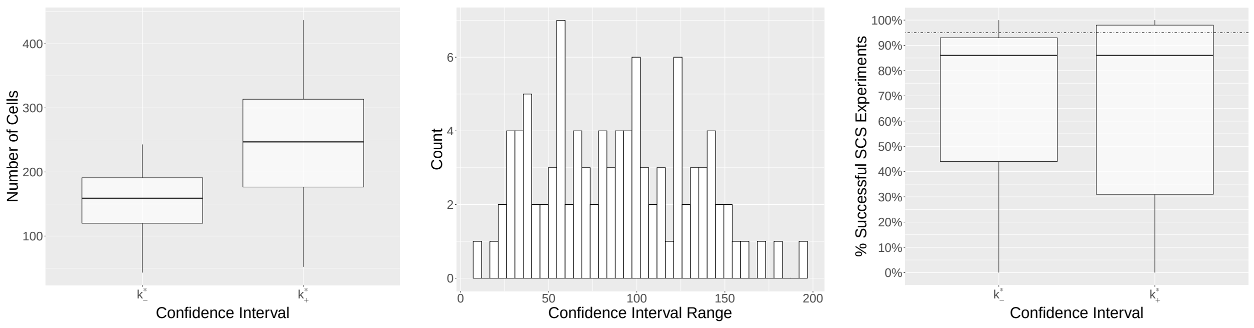

A B C

Fig I: **Computation and evaluation of the k^* confidence interval** (A) Distribution of the number of cells computed by PhyDOSE at confidence level $\gamma = 0.95$ for the k^* confidence interval $[k_-^*, k_+^*]$ in $1m00$ simulations. (B) The distribution of the k^* 95% confidence interval range $(k_+^* - k_-^*)$ over 100 simulations. (C) Distribution of the percentage of successful *in silico* SCS experiments (100 experiments per simulation) when randomly sampling $k \in \{k_-^*, k_+^*\}$ cells computed by PhyDOSE at confidence level $\gamma = 0.95$ (dashed line) in 100 simulations.

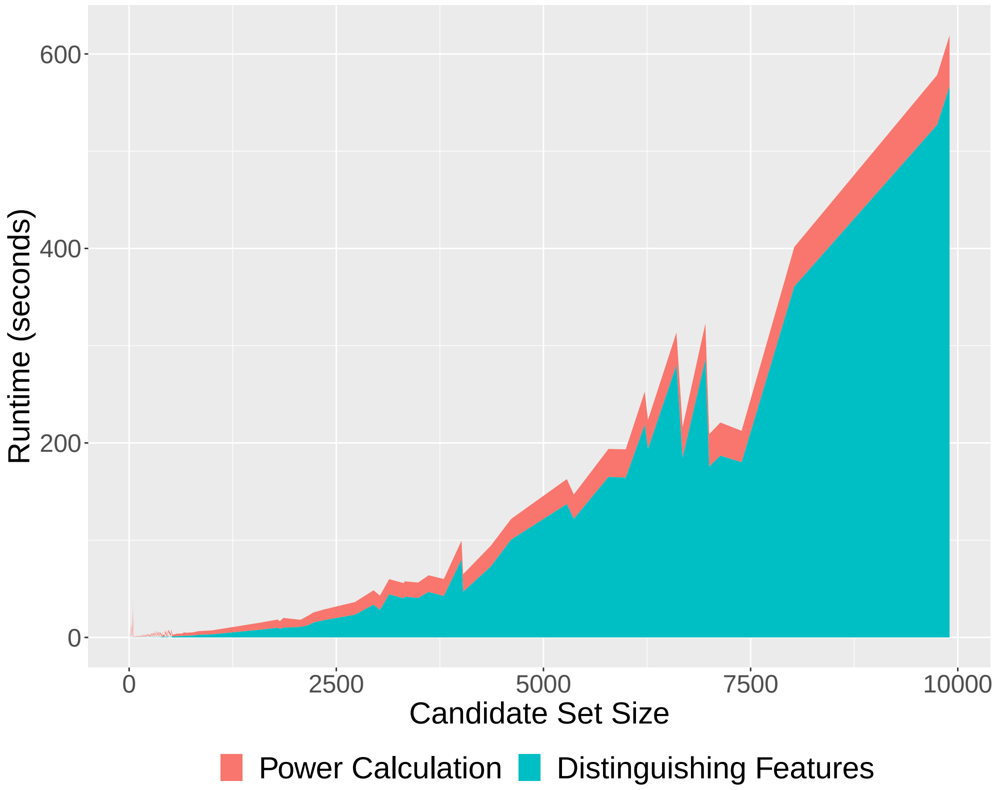

Fig J: **Empirical runtime analysis of PhyDOSE** Total runtime in seconds of PhyDOSE as a function of the input set $\mathcal{T}$ and broken down into the PhyDOSE phases of finding the distinguishing features and then performing the power calculation.

C.1 Retrospective Analysis of an Acute Lymphoblastic Leukemia Patient

For each patient, the sequenced cells are clustered into 2 to 7 clones using an EM-based approach [7]. Based on the fact that false negatives occur more frequently than false positives, we designated an SNV as present if at least 30% of cells in the clone had the mutation. We then checked if the resulting binary, clone-by-SNV matrices adhered to the infinite sites assumption, which was the case for only patients 2 and 3. While the VAFs of all 16 SNVs in patient 2 are less than 0.5, patient 3 had 6 out 49 SNVs with a VAF larger than 0.5, which is indicative of copy number aberrations. Since no copy number information was available to infer cancer cell fractions, we excluded patient 3 from our analysis, thus restricting our attention to patient 2.

For patient 2, 115 cells were clustered into 5 clones [7]. This patient has 16 SNVs, from which we excluded mutations *CMTM8*, *ATRNL1*, *LINC00052* and *TRRAP* for reasons that we described in the main text. The majority voting rule described above yielded a binary clone-by-SNV matrix with 4 mutations clusters that each correspond to SNVs that co-occur in every clone (Fig. Ka), corresponding to a two-state perfect phylogeny T_{SCS} on the mutation clusters ((Main Text) Fig. 5b). To obtain the set $\mathcal{T}$ of candidate phylogenies, we considered the bulk data. Specifically, we merged mutations ZC3H3 and XPO7 as they had the same VAF in the bulk data and occurred in the same mutation cluster in the cleaned SCS data ((Main Text) Fig. 5a). Using SPRUCE [2], we enumerated $|\mathcal{T}| = 2576$ trees (Fig. Kb). Only one tree $T^* \in \mathcal{T}$ was consistent with T_{SCS}, i.e. each mutation cluster of T_{SCS} formed a connected path in T^* and subsequently collapsing these paths in T^* yields T_{SCS}. Comparing the cleaned single-cell data to the raw values, we computed a false negative rate β of 0.2 for the 14 mutations (Fig. Ka), which was in line with the value reported by [7].

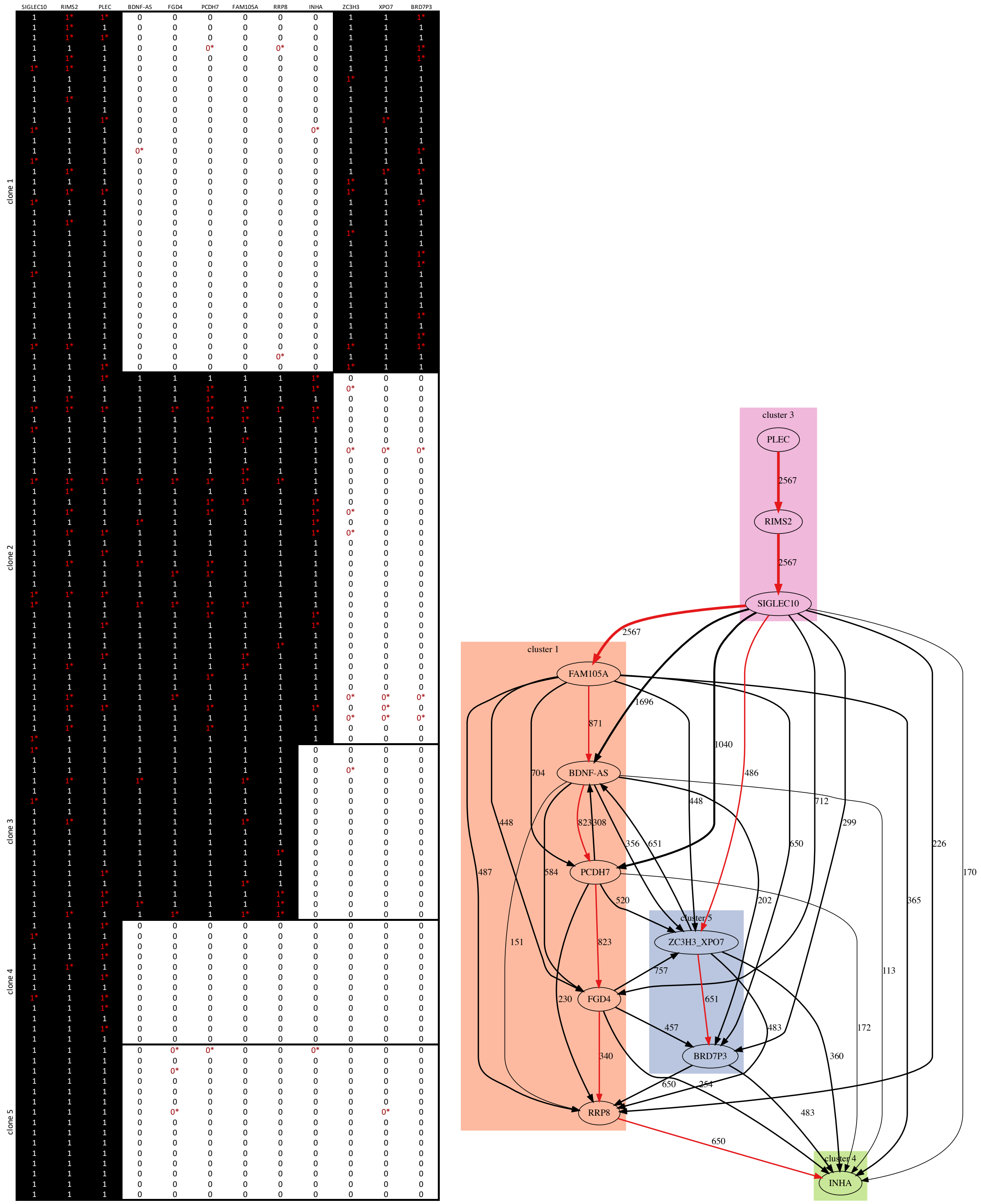

A B

Fig K: **Retrospective analysis of ALL patient 2 where [7] sequenced 115 single cells, targeting 16 mutations.** (A) Overview of single-cell data, focusing on a subset of 14 mutations. Each row is a single cell and columns correspond to mutations. Black cells indicate presence of the SNV, whereas white cells indicate absence. Text in each cell indicates a true positive ('1'), false negative ('1*'), true negative ('0') or false positive ('0*') entry. (B) Overview of the set $\mathcal{T}$ of trees, where each edge is labeled by its frequency in $\mathcal{T}$. Red edges encode the tree T^* that is consistent with SCS data in (a). In particular, the SCS data encodes a tree composed of four mutation clusters, each indicated by a different shading.

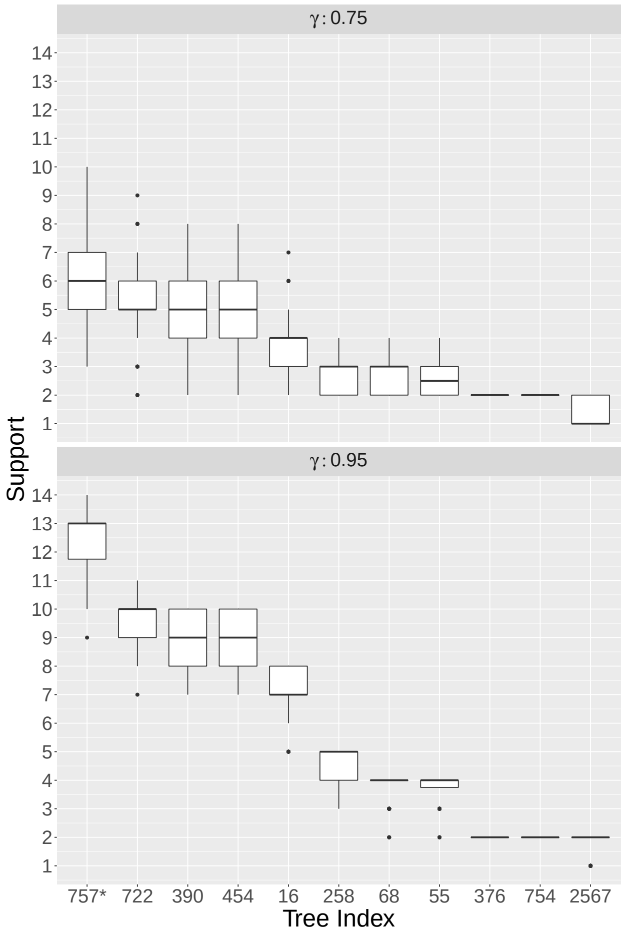

Fig L: **Distribution of number of supporting cells for each candidate tree across 100 *in silico* SCS experiments at varying success probabilities for ALL patient 2 [7].** For $\gamma = 0.75$, we sampled $k(T^*) = 50$ cells from the SCS data. For $\gamma = 0.95$, we sampled $k(T^*) = 103$ cells. Fig. M shows the candidate trees.

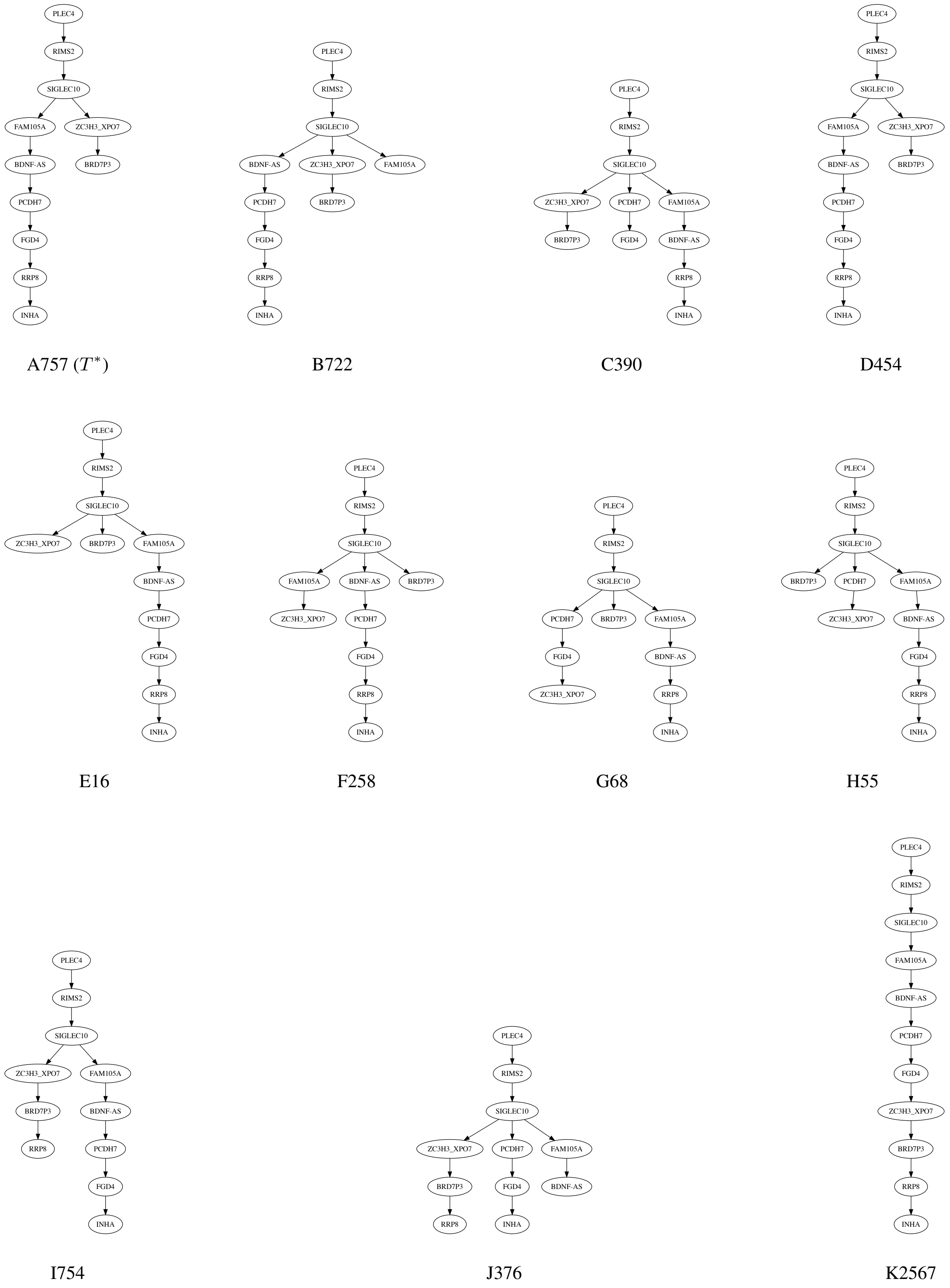

Fig M: **Candidate trees that had a non-zero support across 100 *in silico* SCS experiments for ALL patient 2 [7].** For $\gamma = 0.75$, we sampled $k(T^*) = 50$ cells from the SCS data. For $\gamma = 0.95$, we sampled $k(T^*) = 103$ cells. Labels match tree indices in Fig. L.

patient	mutations	total sequenced [8]	$\|\mathcal{T}\|$	$k^*_{\gamma=0.75}$ (% red.)	SCOPIT $\gamma = 0.75$	$k^*_{\gamma=0.95}$ (% red.)	SCOPIT $\gamma = 0.95$
AML-4-001	5	5878	8	451 (92.3%)	505	973 (83.4%)	973
AML-5-001	4	10191	8	396 (96.1%)	1156	730 (92.8%)	2463
AML-14-001	3	7053	4	129 (98.2%)	574	278 (96.1%)	1018
AML-22-001	6	2925	57	209 (92.9%)	5322	402 (86.3%)	11500
AML-26-001	5	9601	24	306 (96.8%)	489	590 (93.9%)	916
AML-29-001	3	7795	2	78 (99.0%)	78	169 (97.8%)	169
AML-30-001	3	6668	2	83 (98.6%)	605	178 (97.3%)	1307
AML-32-001	4	8241	2	7 (99.9%)	75	14 (99.8%)	161
AML-36-001	3	8153	2	246 (97.0%)	256	531 (93.5%)	531
AML-38-001	8	7235	316	366 (95.0%)	5424	692 (90.4%)	10794
AML-39-001	4	6735	6	56 (99.2%)	101	120 (98.2%)	193
AML-40-001	3	6194	2	60 (99.0%)	60	128 (98.0%)	128
AML-41-001	7	5082	89	413 (91.9%)	34837	1155 (83.0%)	75281
AML-42-001	3	2252	2	125 (94.4%)	125	270 (88.0%)	270
AML-45-001	3	7716	2	29 (99.7%)	30	62 (99.2%)	62
AML-51-001	3	8219	2	216 (97.3%)	216	467 (94.3%)	467
AML-59-001	3	2662	2	41 (98.5%)	41	88 (96.7%)	88
AML-61-001	4	4320	2	64 (98.5%)	352	138 (96.8%)	643
AML-62-001	3	4207	2	9 (98.5%)	27	20 (99.5%)	55
AML-64-001	4	4999	3	225 (95.5%)	301	471 (95.5%)	550
AML-67-001	5	6024	2	21 (99.7%)	55	44 (99.2%)	91
AML-69-001	3	7462	2	202 (97.3%)	202	436 (94.2%)	436
AML-75-001	3	6073	2	20 (99.7%)	50	42 (99.3%)	102
AML-76-001	4	8033	3	196(97.5%)	211	423 (94.7%)	423

Table A: **Prospective analysis of an acute myeloid leukemia (AML) cohort.** Table shows the patient identifier, the number of mutations,the total cells sequenced [8], the size of the candidate set $\mathcal{T}$ of trees as determined by SPRUCE [2], PhyDOSE's k^* calculated at confidence levels of $\gamma \in \{0.75, 0.95\}$ (% reduction from total sequenced), and comparison with SCOPIT at confidence levels of $\gamma \in \{0.75, 0.95\}$.

patient	clusters	samples	min muts	max muts	$\lvert\mathcal{T}\rvert$	k^* single sample	k^* mul. sample	sel. sample
CRUK0004	7	4	10	78	2	32	32	R3
CRUK0005	6	4	24	536	2	26	26	R3
CRUK0011	8	3	12	335	3	34	34	R2
CRUK0012	5	2	11	84	2	15	15	R1
CRUK0013	9	5	5	114	8	1051	215	LN2=172<br>R3=43
CRUK0022	5	2	5	158	2	23	23	R1
CRUK0023	10	4	5	226	2	20	20	R1
CRUK0025	7	3	10	350	2	25	25	R2
CRUK0028	5	2	5	72	2	33	33	R1
CRUK0031	7	3	15	675	2	30	30	R1
CRUK0037	10	5	6	397	17	∞	234	R1=36<br>R2=49<br>R3=60<br>R4=38<br>R5=51
CRUK0038	4	2	6	107	2	43	43	R1
CRUK0046	5	4	5	186	2	29	29	R1
CRUK0049	6	2	7	882	4	37	37	R2
CRUK0063	8	5	5	167	2	33	33	R4
CRUK0067	5	2	24	263	2	29	29	R1
CRUK0068	10	4	11	532	3	35	35	R2
CRUK0070	10	5	6	254	2	24	24	R6
CRUK0076	9	4	8	846	4	47479	48	R1=23<br>R2=25
CRUK0077	7	4	9	586	2	22	22	R4
CRUK0084	6	4	5	332	2	17	17	R2
CRUK0094	6	4	6	50	2	22	22	R4
CRUK0095	4	3	6	216	2	42	42	R2
CRUK0099	5	4	5	438	2	46	46	R6
CRUK0100	8	3	5	777	3	24	24	R2

Table B: **Prospective analysis of TRACERx non-small-cell lung cancer cohort with confidence level $\boldsymbol{\gamma = 0.95}$.** Table shows the patient identifier, the number of mutation clusters, the number of bulk samples, the minimum number of mutations per cluster, the maximum number of mutations per cluster, the size of the candidate set $\mathcal{T}$ of trees as determined by [9], PhyDOSE's k^* calculated at confidence levels of $\gamma = 0.95$ for the minimum single sample and the multiple sample heuristic and the recommended sample label from which the single cells should be drawn.

References

[1] Karp RM. In: Miller RE, Thatcher JW, Bohlinger JD, editors. Reducibility among Combinatorial Problems. Springer; 1972. p. 85–103.

[2] El-Kebir M, Oesper L, Acheson-Field H, Raphael BJ. Reconstruction of clonal trees and tumor composition from multi-sample sequencing data. Bioinformatics. 2015;31(12):i62–i70.

[3] El-Kebir M, Satas G, Oesper L, Raphael BJ. Inferring the Mutational History of a Tumor Using Multi-state Perfect Phylogeny Mixtures. Cell Systems. 2016;3(1):43–53.

[4] El-Kebir M, Satas G, Raphael BJ. Inferring parsimonious migration histories for metastatic cancers. Nature Genetics. 2018;50(5):718–726.

[5] Davis A, Gao R, Navin NE. SCOPIT: sample size calculations for single-cell sequencing experiments. BMC Bioinformatics. 2019;20(1):566.

[6] El-Kebir M. SPhyR: tumor phylogeny estimation from single-cell sequencing data under loss and error. Bioinformatics. 2018;34(17):i671–i679.

[7] Gawad C, Koh W, Quake SR. Dissecting the clonal origins of childhood acute lymphoblastic leukemia by single-cell genomics. Proceedings of the National Academy of Sciences. 2014;111(50):17947–17952. doi:10.1073/pnas.1420822111.

[8] Morita K, Wang F, Jahn K, Kuipers J, Yan Y, Matthews J, et al. Clonal Evolution of Acute Myeloid Leukemia Revealed by High-Throughput Single-Cell Genomics. bioRxiv. 2020;doi:10.1101/2020.02.07.925743.

[9] Jamal-Hanjani M, Wilson GA, McGranahan N, Birkbak NJ, Watkins TB, Veeriah S, et al. Tracking the evolution of non–small-cell lung cancer. New England Journal of Medicine. 2017;376(22):2109–2121.
