# Supplementary material for: PhyDOSE: Design of follow-up single-cell sequencing experiments of tumors
Source: PLoS Comput Biol. 2020 Oct 1;16(10):e1008240. doi: 10.1371/journal.pcbi.1008240 (PMC7553321; doi:10.1371/journal.pcbi.1008240)
Supplement: Attachment — Submitted filename: response.pdf [file pcbi.1008240.s002.pdf]

We thank the reviewers for their extensive feedback. In summary, the main concerns were about comparisons with other methods (reviewer #3) and practical limitations (reviewer #1, #2). We have addressed these concerns as follows:

1. We have included a comparison against SCOPIT to demonstrate the advantages of our method, including a reduction in the number of cells to sequence.
2. We have addressed concerns regarding uncertainty in bulk frequency estimation by developing a methodology to find a confidence interval on the number of cells to sequence while accounting for the uncertainty of frequency estimates.
3. To address the reviewers' comments on the increased availability of multiple biopsies and the importance of considering different spatial regions to capture heterogeneity, we formulated two new problems, the Multi-Sample SCS Power Calculation (Mul-SCS-PC) and the Multi-Sample SCS Power Calculation for Phylogeny T (T-Mul-SCS-PC) and provide a method to obtain an exact solution as well as a heuristic that scales for practical problem sizes. We demonstrated the utility of this addition by applying it to a lung cancer cohort in which multiple biopsies are available.
4. Finally, we have included an additional experiment to demonstrate that PhyDOSE is robust to violations of the infinite sites model.

Please find below our detailed point-by-point responses in blue font.

Reviewer #1:
This paper develops a method, PhyDOSE, for designing follow-up single-cell sequencing experiments to resolve tumor phylogenies based on initial bulk sequence analysis. This paper is based on a conference submission that has already been through a round of revision in response to the conference reviewers. Although I was not one of the reviewers of the conference version, I recognize that it is more polished than a typical new submission as a result and that the conference reviewers already raised what would have been some of my concerns about the paper. I therefore read it in the spirit of a paper that has already been through a round of revisions. The general problem is well motivated. Although the cost of single-cell sequencing is dropping rapidly, it remains costly for large studies and there is good reason to use it efficiently. The approach proposed here appears sensible and technically sound, making good use of prior theory of the authors on tumor phylogeny enumeration, adding robust handling for common kinds of data errors in single-cell sequencing, and connecting the theory nicely to a rigorous new probabilistic model for use in power calculations. The method is well backed up by empirical analysis of simulated data and three real cohorts, showing the method to be effective and to improve substantially on naïve approaches in at least some cases. Some natural concerns about the approach in the conference version, such as robustness to biased sampling or to failing to identify some clones, were raised by the conference reviewers and effectively rebutted by new experiments. The method does still make some restrictive assumptions and limitations, as was pointed out by the prior reviewers. While these are not fully resolved even in the revision, the authors do have fair responses for them.

Given this, I have little to add in the way of criticism and would consider my remaining points largely discretionary. My only real substantive concern is that some of the limitations of the method raised in the conference reviews are still limitations and one might question whether it is sufficient in some cases to note them and defer them to future work. I refer here essentially to the points raised in the final paragraph of Discussion.

In that regard, the use of the infinite sites model is questionable enough that one could argue it needs to be at least demonstrated that the method is reasonably robust to violations. While many methods in this space use the infinite sites assumption, it is well established that it is not consistently accurate and is at least becoming more accepted that methods must handle some violations. I think it is fine to defer to future work extension to a more robust model like Dollo parsimony, which would understandably require some significant changes to the theory and algorithms, so long as the method works reasonably well on data that violates the assumption without that.

Thank you for raising this point as it is a very valid concern that could impact its practical use. To evaluate PhyDOSE under violations of the infinite sites assumption (ISA), we performed an additional experiment on a candidate tree set with a mutation loss (sim1a 1-Dollo). In Results, we demonstrate that PhyDOSE's computed number of cells leads to uniquely and correctly identifying the true phylogeny in the majority of cases.

The paper also considers a criticism about the use of single rather than multiple bulk samples and defers that question to future work. There is good evidence that phylogeny inference from single bulk samples is simply not accurate enough to be the basis for even the initial step of a combined bulk and single-cell study, and so one might reasonably argue that accommodating multiple bulk samples is so important that it should be part of even a first method of this class.

Yes, we agree with the reviewer's point here and therefore in our revised manuscript we introduce two new problems, the Multi-Sample SCS Power Calculation (Mul-SCS-PC) and the Multi-Sample SCS Power Calculation for Phylogeny T (T-Mul-SCS-PC) to address the availability of multiple bulk samples. We provide a method of calculating the solution to the T-Mul-SCS-PC but since this solution does not scale to realistic problem sizes, we also offer a heuristic that conservatively estimates the number of cells to sequence. We use this approach to recompute the number of cells to sequence for the lung cancer cohort and compare it with the naive approach of treating each sample independently. We show that in the case of three patients in the cohort, using the multiple sample heuristic can lead to a significant reduction in the number of cells to be sequenced compared to the naive approach. Specifically, at a confidence level of 0.95, one patient (CRUK0076) would have required 47,479 cells in the best case if the sample were treated independently and only 48 cells if selected across two of the available samples.

I will also just raise as a discretionary thought some other possible scenarios where I could imagine this method being useful. I wonder if the method could be applied if there has already been some bulk and some single-cell sequencing done, as in some studies to date, and we want to plan further single-cell sequencing. Would the method be adaptable to such a case? Or could

it do better if we assume multiple batches of single-cell sequencing, with an opportunity to reevaluate after each batch? I can accept that these are getting far enough afield that they do not need to be solved in this paper, but might also be questions for future work.

This is a very good point and we agree that making use of all available data or even obtaining some preliminary single-cell data prior to designing an experiment could improve the final inference results. If single-cell data in addition to or perhaps instead of bulk data is available, then the set of candidate trees could be formed from either a posterior distribution of a Bayesian inference method or multiple optimal solutions from a combinatorial approach. As long as the input is a set of candidate trees and there exists a way to estimate the clonal prevalence of each clone in the distinguishing features, then PhyDOSE can still be utilized in any of these scenarios. We facilitate this mode in our R package phydoser by allowing direct input of the clonal prevalence rates, which may be obtained from single-cell inference methods. In Discussion, we now write:

"Although PhyDOSE is motivated by the output of deconvolution methods for bulk sequencing, it is agnostic to the method used to obtain the candidate set as long as the clonal prevalence rates of the distinguishing features can be estimated. Thus, the input set $\mathcal{T}$ of candidate trees can be obtained from preliminary single-cell and/or bulk sequencing data." (lines 607-611)

Reviewer #2:
This paper discusses PhyDOSE, a method to perform power calculation for single-cell sequencing, when we need to disentangle the clone tree associated to a tumour sample. The idea is that, while we often perform a bulk sequencing experiment to assess a number of possible trees that fit the mutation allele frequency (VAF), it happens often that more than one tree are equally-likely to fit the data. If we can generate single-cell sequencing data of a number X of single-cells, then we can disambuiguate which tree best fits the data. PhyDOSE is a method that tells us what should be the value of X to bound the probability that we can determine a unique best tree.

The paper is clear, and the problem is known in the field. There is abundant literature explaining/ showing that determining a single tree from bulk data can be challenging, therefore the solution of sequencing single cells can be appealing, as much as other approaches. The ILP formulation of the problem seems to be correct, and the results and methods consistent with the theory.

However, there are some major limitations of the work in the current form. I think fixing them would make the main message (a computational design technology) appealing.

- I do not think that you can prescind from the fact that many datasets collect multiple tumour bulks at once, as you also note in your Discussion. This requires a multivariate problem definition, in principle, that you need at least to discuss. There are multi-region sequencing simulators that you can use to this respect, if you want to try to simualte data. The current work presents instead an independence assumption, and uses that in the current analyses (sect

"Prospective Analysis of a Non-small Cell Lung Cancer Cohort"). The choice of PhyDOSE is to minimise the number of cell estimates across all samples; is this supported by some consideration?

Thank you for raising this excellent point. We agree and have addressed this limitation by introducing two new problems: the Multi-Sample SCS Power Calculation (Mul-SCS-PC) and the Multi-Sample SCS Power Calculation for Phylogeny T (T-Mul-SCS-PC) when multiple bulk samples are available and offering an exact calculation, which does not scale, and a heuristic to solve the problem in practice. Please see our above response to Reviewer #1 on this point for additional details.

- [related to the above] what do the author mean by saying that "Mutation clusters alleviate the issue of false negatives, i.e. it suffices to only observe a single mutation to impute the presence of the other mutations in the same cluster.". Imputation can be tricky; if I observe a low-VAF mutation in 3 out of 4 biopsies, I think that the imputation should depend on the coverage at the locus. If high-enough, imputation can be supported by a statistical argument based on Binomial testing on read counts (what are the odds of not-seeing a mutation with a certain VAF with my current coverage). If low, imputation might generate false positives. Is it possible to frame this uncertainty in PhyDOSE's computation of the optimal number of cells for these scenarios?

Yes, we agree with the reviewer. We now write:

"Assuming high confidence on the co-occurrence of mutations in a cluster, mutation clusters alleviate the issue of false negatives, i.e. it suffices to only observe a small number of mutations to impute the presence of the other mutations in the same cluster." (lines: 577-579)

To further address the important issue of uncertainty in VAF estimates, we have developed a method to obtain a confidence interval for PhyDOSE's output k*. The goal of this confidence interval is to bound k* for a given tree by the **largest possible value required** if the actual VAF estimates assume values yielding the smallest possible clonal prevalence of a clone in the distinguishing feature, and **the smallest possible k*** when VAF estimates assume values yielding the largest possible clonal prevalence of the smallest clone in the distinguishing feature. We include simulations to assess this new functionality of PhyDOSE.

- You present some reduction in cells numbers that are not exceptionally striking. Can you justify a difference in sequencing cost for the effort of using your design method? At the end of the day, if one does not save a substantial amount of sequencing costs, why would he/ she bother using PhyDOSE? I think you need to provide stronger evidence of why your computations can be important for a molecular biologist that is designing a new experiment. If the reduction is not substantial, I think that your contribution would be just theoretical and could be less appealing. In the context of sequencing technologies, you should put effort to understand the cost for standard experimental setups (e.g., I presume you would be using either a deep sequencing panel, or a digital-PCR assay) and their possible parametrisation. On

your real data you can effectively discuss these reductions (assuming certain costs since you did not generate the data).

We thank the reviewer for this point. We showcased the decrease in sequencing costs achieved by PhyDOSE in the AML cohort, where single-cell sequencing was performed with a targeted panel using the Tapestri platform. We write:

"*Morita et al. [27] sequenced 153,558 cells while the PhyDOSE design at confidence level gamma = 0.95 requires 8,144 cells (S1 Table). Using that a Tapestri run of 10,000 cells costs $795 and including additional sequencing costs of $200 per run with NovaSeq or $1000 per run with MiSeq [39, 40], we estimate the costs of the original study as $15,920 and the estimated costs of the PhyDOSE design as $1,995. This assumes the original study utilized 16 Tapestri runs with NovaSeq while PhyDOSE requires 1 Tapestri run with the more expensive MiSeq to avoid multiplexing. Thus, designing the experiment with PhyDOSE would have yielded a 93,75% cost savings. Further, we note that this study design requires targeted sequencing and that the potential cost savings when the experimental design requires whole genome sequencing would further increase.*" (lines: 557-567)

Further, we note that the main goal of PhyDOSE is not to reduce the costs of the experiment but instead to provide a rigorous framework to guide the experimental design when the goal is phylogeny inference. Hence, we say PhyDOSE yields cost efficient experimental designs because we find the smallest number of cells to be sequenced such that the experiment is sufficiently powered at a given confidence level.

- It is increasingly evident that a number of "clusters" identified through standard VAF deconution method can represent random ancestors constituted by neutrally evolving mutations (https://doi.org/10.1101/586560). Clustering tail mutations is also wrong becauoe tail lineages are polyphyletic. You should discuss this when you consider the problem of using certain subsets of mutations. Since some of the input clusters should be removed from the clone trees, and you could discuss what happens if you end up taking cells from those clusters to design your experiment. This is important because many of your inconsistencies in assembling a bulk clone tree stem from low-frequency mutations, but the low-VAF spectrum is where most of the neutral mutations reside; if those are removed how often is it that you remain with a non-identifiable treee?

The point that you raise actually occurred in our analysis of Patient 2 in the ALL cohort so we will describe our approach along with our reasoning. Prior to removing low-VAF mutations, deconvolution methods yielded a candidate set size of 2.5 million trees. As described in the Main Text, we opted to exclude low-VAF mutations (<= 0.05), which resulted in a candidate set size of 2,576:

"*Using SPRUCE [9], we enumerated the set $\mathcal{T}$ of trees from the bulk data, yielding over 2.5 million trees. This number is mainly driven by 3 mutations (ATRNL1, LINC00052 and*

TRRAP) with a VAF less than 0.05. Excluding these 3 mutations resulted in a more tractable number of 2,576 trees." (lines 508-511)

Moreover, our simulations had large sets of candidate trees (median: 59) while specifically enforcing mutations to have a VAF of at least 0.05:

"We used rejection sampling to ensure that each clonal prevalence u_i was at least 0.05." (lines 330-331)

Reviewer #3:
The authors report on a new method, PhyDOSE, for determining the number of cells to sequence in a single-cell sequencing experiment based on information from bulk data. The bulk data is first used to estimate the mutation frequencies and then this information is used to estimate the number of cells. The authors state that their method improves upon SCOPIT, since the latter assumes knowledge about the number of clones and the frequency of the smallest clone. The authors study the performance of their method on simulated and empirical datasets.

I would like the authors to address the following questions:
1. How does the reliability of the mutations called from the bulk data affect the performance of the method? What if some/many of those mutations were wrong?

As mentioned in the response to Reviewer #2, we agree that this is an important point to address in this work. To consider uncertainty in mutation calls and VAF estimates, we developed a method to obtain a confidence interval for k*. For more details, please see the related response to Reviewer #2's comments.

2. Why not compare to SCOPIT? After all, there are method for estimating clonality from bulk data. Why not run such a method, get the number of clones and frequencies, and use those as inputs to SCOPIT? I think it's very important to do this comparison.

In our revised manuscript, we have now included a comparison with SCOPIT. In order to compare against SCOPIT, we require estimates of the clonal prevalence rates of the clones as was pointed out in the above review. Therefore, we compare against SCOPIT in two modes. First, we consider uncertainty in the clones to be observed by using SCOPIT on each tree in the candidate set and taking the upper bound, similar to PhyDOSE. However, to additionally compare against SCOPIT in its optimal use case, we give SCOPIT the clonal prevalence rates of the ground truth simulated tree. We show that by relying on the distinguishing features of a tree, PhyDOSE significantly outperforms SCOPIT in the case of clone uncertainty and even outperforms SCOPIT in the best case for the majority of simulation replications. This is because PhyDOSE always considers a subset of the clones considered by SCOPIT. To demonstrate this, we also compare the PhyDOSE k* of the ground truth tree to SCOPIT and show that the number of cells to sequence is always less than or equal to SCOPIT.

3. I think the model of evolution must be incorporated into the problem formulation, as the number of cells and mutations needed depends on whether the infinite-sites assumptions holds or not.

As suggested by Reviewer #1, we have included an additional experiment that evaluates a candidate set of 1-Dollo phylogenies. The experiments show that because the distinguishing features provide an efficient representation of each tree in the set, PhyDOSE performs well on data that violates the presumed ISA model of evolution. Please see our above response to reviewer #1 for additional details. However, in the cases when the distinguishing features of the true tree are impacted by the mutation loss, PhyDOSE performs poorly. As future work, we plan to develop a more careful approach to find the distinguishing features of a tree under a wider array of evolutionary models.

My main issue with this method (which applies to SCOPIT as well, I feel, even though I don't know the details of how SCOPIT works) is that the number of cells to sequence is not the only/main quantity of interest in in an SCS experiment. The number of spatial regions to sample and sequence in order to capture the heterogeneity is as important, and that number must be a lower bound on the number of cells to sequence. So, I'm not sure how useful these methods will be in practice. Yes, SCOPIT has been published for a year only, but it still has no real citations (the two citations it has are this article and one that develops a simpler method for scRNA data). As scDNAseq becomes even less expensive, I doubt the number of cells is the bottleneck; it's the spatial regions to sample and sequence (indeed, some recent studies, mainly focused on CNA detection, are now sequencing thousands of single cells).

With the inclusion of the multiple biopsy heuristic, PhyDOSE now provides additional guidance on which of the available biopsies should be sequenced and how many cells to sequence from each biopsy. For example, in the TRACERx lung cancer cohort, PhyDOSE returned infinity when requiring that all cells be sequenced from a single biopsy. Using the multiple biopsy heuristic, we not only obtain a finite number of cells (234) but specifically denote how many cells should be sequenced from each of the five available biopsies. When multiple samples are available, this heuristic helps to design experiments that better capture tumor heterogeneity.

Two other recent papers (Su et al. 2020 [doi: https://doi.org/10.1093/bioinformatics/btaa607], Liang et al. 2020 [doi: https://doi.org/10.1101/2020.05.31.126565]) seek to address related challenges in scRNA-seq experiment design, which leads us to suspect that there is growing interest in this topic. Perhaps one reason why SCOPIT did not garner a lot of citations in its first year is because it presumes the user knows the clonal prevalence rates of the clones to be observed. This is unlikely in practice when the goal of the experiment is phylogeny inference. Thus, SCOPIT seems to be most useful when trying to confirm the accuracy of a high confidence phylogeny or set of clones. In contrast, PhyDOSE is a method to help resolve uncertainty of the clones/phylogeny. We hope that by demonstrating a substantial cost-savings for the AML cohort of $14,000, in which targeted sequencing was used, researchers will be more motivated to make strategic use of their sequencing budgets.

We agree with the reviewer that the number of cells is not the only quantity of interest. As mentioned in the Discussion, we plan to study the problem of selecting a subset of mutations to target. There are other exciting future directions, including the design of SCS experiments that use multiple whole genome amplification strategies (MDA and DOP-PCR) to facilitate reconstruction of tumor phylogenies that include both single-nucleotide variants and copy number aberrations. We believe that this work will provide a solid foundation for these future directions.
